# Nailfold capillary patterns correlate with age, gender, lifestyle habits, and fingertip temperature

**DOI:** 10.1371/journal.pone.0269661

**Published:** 2022-06-15

**Authors:** Tadaaki Nakajima, Shizuka Nakano, Akihiko Kikuchi, Yukiko T. Matsunaga

**Affiliations:** 1 Institute of Industrial Science, The University of Tokyo, Tokyo, Japan; 2 Department of Materials Science and Technology, Tokyo University of Science, Tokyo, Japan; National Taiwan University Hospital, TAIWAN

## Abstract

Nailfold capillaroscopy is a simple and noninvasive imaging tool to visualize the pattern of capillaries. Microvascular abnormalities have been previously observed in autoimmune disease such as systemic sclerosis and diabetes. Thus, early detection of microvascular dysfunction or changes has promising way for the one of the disease preventions. In this study, for routine health checkups, we evaluated the relationship between the structure of nailfold capillaries and lifestyle habits in healthy participants. First, we analyzed the correlation of structural parameters of nailfold capillaries with values of responses to questions on their lifestyle habits in 224 participants. The results suggested that an unhealthy lifestyle, including poor sleeping habits, smoking, intense exercise, and drinking alcohol, causes a change in the pattern of nailfold capillaries. We then investigated whether the pattern of nailfold capillaries changed after a conscious improvement in lifestyle habits. One to two weeks after the self-improvement of lifestyle habits, the hairpin loops sharpened or straightened. In conclusion, this study is the first report indicating a correlation between the structure of nailfold capillaries and lifestyle habits in a non-clinical population. The simple, inexpensive, and noninvasive method using nailfold microscopy can be employed for routine health checkups everywhere even at a bedside.

## 1. Introduction

In the past 28 years, the rate of healthy life expectancy in overall life expectancy decreased from 86.9% in 1990 to 86.7% in 2017 [1]. The difference between healthy and overall life expectancy increased by >1 year in most countries, including countries with high and low socio-demographic index [1]. The smaller increase in healthy life expectancy than the increase in overall life expectancy is a global concern. Poor habits can cause cardiovascular diseases [2], cancer [3], diabetes [4], and Alzheimer’s disease [5]. Healthy lifestyle changes, including exercising and reducing psychological stress, smoking, and alcohol intake, can prevent diseases. For routine health checkups, simple, inexpensive, and noninvasive methods are constantly being developed.

Blood vessels are closely related to numerous diseases. Blood vessels are present in almost all tissues in the body, and perform numerous physiological roles, including transportation of gases, heat, nutrition, metabolites, and immune cells to all tissues [6]. The vasculature is organized into a hierarchical network of arteries, veins, and interconnected capillaries, with a diameter of <50 μm [7]. Capillaries form a network in which most intercellular communication to tissues occurs [6]. The structure of the capillary changes abnormally in cardiovascular diseases, obesity [8], cancer [9], Alzheimer’s disease [10], and diabetes [11]. Thus, the relationship between blood vessel structure and disease has led researchers to develop noninvasive observation systems for the early detection of disease.

Nailfold capillaroscopy is a noninvasive detection method for routine medical check-up. In the latter part of the 18th century, Giovanni first noted the relationship between conjunctival inflammation and the presence of a cross point of nailfold capillaries using a magnifying glass [12]. Since the 20th century, nailfold capillaries have been extensively observed using a microscope, which has demonstrated a relationship between the structure and diseases [12, 13]. Owing to advances in digital imaging technology, analytical methods have been established to evaluate structural parameters (e.g., the diameter and density of capillaries) [14–16]. The pattern of nailfold capillaries correlates with various types of disease (mainly autoimmune disease) [17–22], and recently the correlation with diabetes has been reported [23, 24]. Furthermore, nailfold capillaroscopic analyses have high sensitivity and specificity in detecting primary Raynaud’s phenomenon presenting cold fingertip, and systemic sclerosis-related interstitial lung disease [13, 25]. The structural changes in the nailfold capillary has been reported in the unhealthy lifestyle habits such as smoking [26], etc. and even psychological stress presents slow blood flow [27]. Therefore, study in the relationships between the structures of nailfold capillaries and lifestyle habits has high potential to understand the unhealthy state, or even predict the pre-disease condition. The normality or normal range of the nailfold capillaries has been investigated in the healthy individuals in terms of structure [28]. In this study, we have evaluated the relationship between nailfold capillary pattern and lifestyle habits and coldness of the fingertips which may exhibit microcirculation disorder linked with pre-disease.

## 2. Materials and methods

### 2–1. Study design and participants

**Fig 1:**
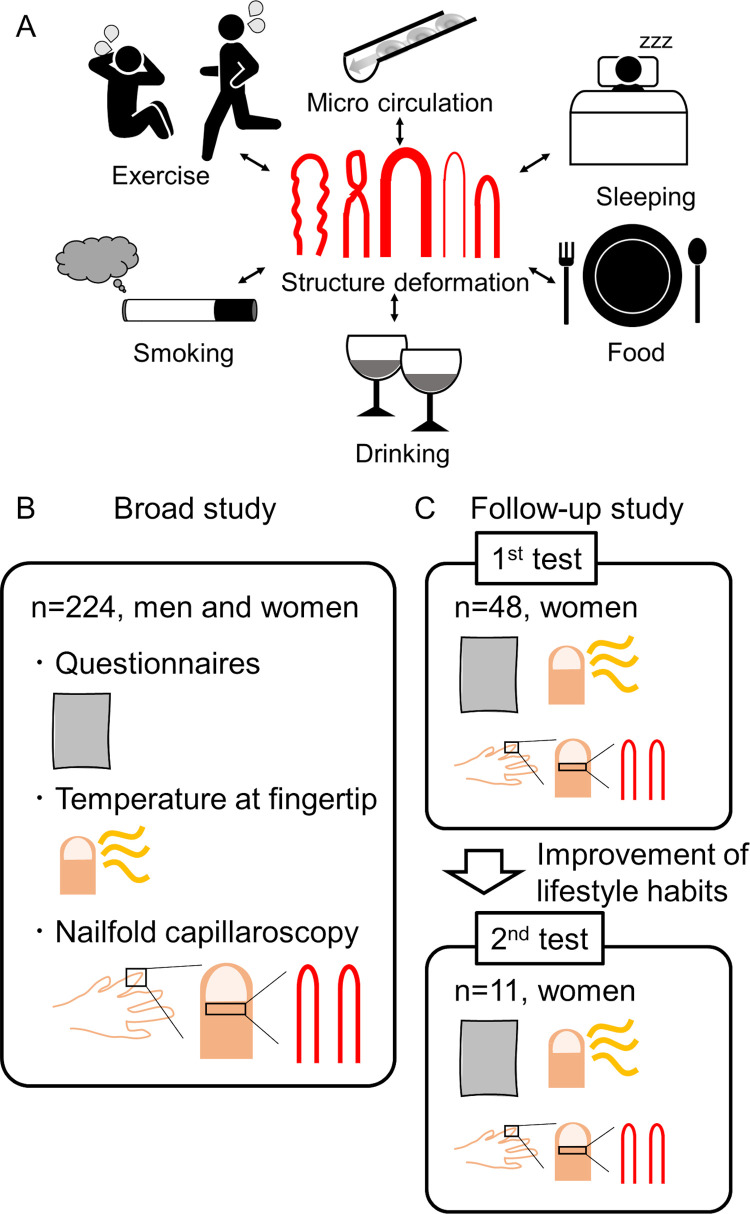
Study design. A: Schematic representation of the study to evaluate the correlation between pattern of nailfold capillaries and lifestyle habits. Clinical experimental design for the broad study (B) and follow-up study (C). In the broad study, both men and women participated, and data were collected as questionnaires for lifestyle habits, fingertip temperatures, and images using nailfold capillaroscopy. In the follow-up study, women participants addressed the improvement in lifestyle habits after the first test, and data were reanalyzed after 1–2 weeks.

These studies were exploratory tests, and comparisons between parameters of nailfold capillary and lifestyle habits served as our primary aim. We conducted a broad study and a follow-up study, as illustrated in Fig 1A. The exclusion criteria of participants for analysis were a history of cardiovascular and immune disease and medications, including diabetes.

#### Broad study

We recruited 224 participants (62 men aged 20–39 years, 38 men aged 40–79 years, 65 women aged 20–39 years, and 59 women aged 40–69 years) of Asian ethnicity (>90% Japanese men and women) by voluntary response in 2018 June. Inclusion criteria of participants were participants who aged ≥20 and can decide to participate the clinical test by themselves. The temperature of the fingertips of the observed finger was measured using an infrared thermometer (IR-301; CUSTOM, Tokyo, Japan).

### 2–2. Questionnaires

To investigate the lifestyle habits of the participants, we prepared questionnaires including questions on sex, age, frequency of coldness of fingertip, sleep quality, time taken to fall asleep, intense exercise, eating oily food, drinking alcohol, and smoking, which were answered using a number between 1 (low) and 5 (high). A list of the questions from both studies is presented in S1 Table of S1 File.

### 2–3. Nailfold capillaroscopy

**Fig 2:**
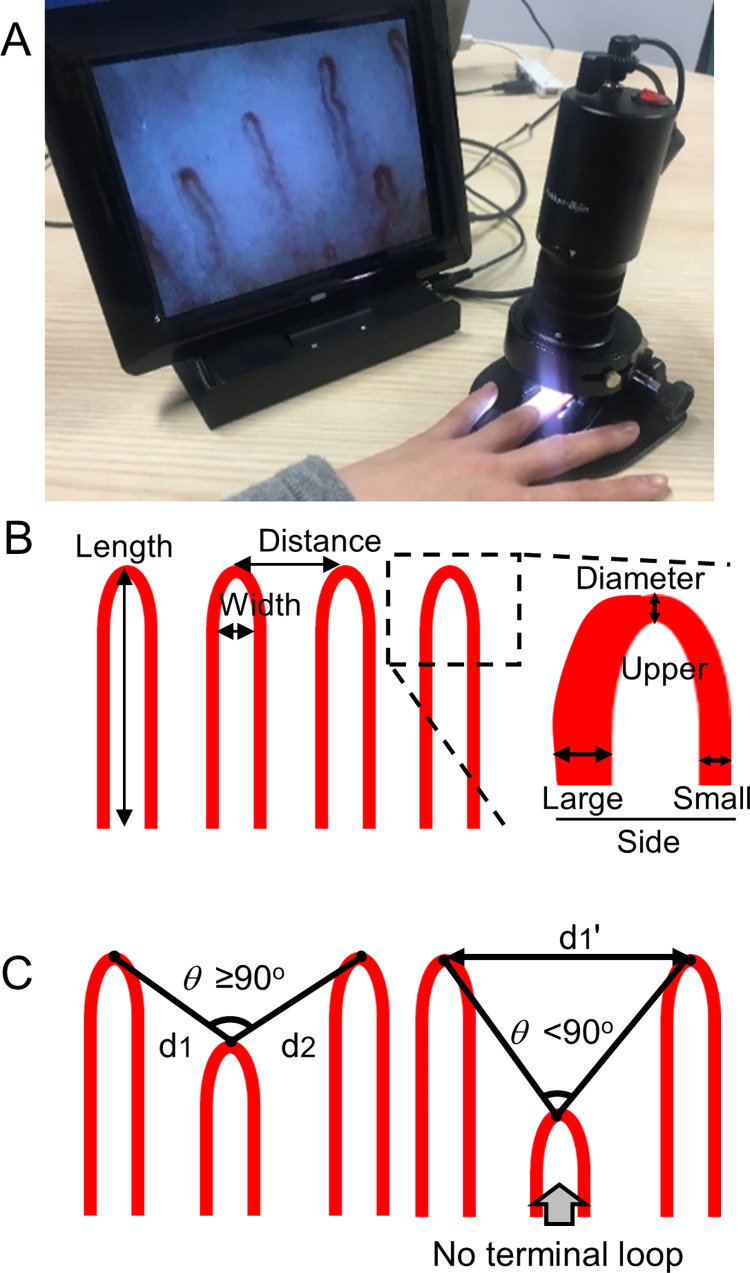
A method for evaluating the structure of nailfold capillaries. A: The light microscope used for observing nailfold capillaries. B: The parameters used for determining the structure of nailfold capillaries in this study. Length of the capillary loop from bottom to apex point, width of the parallel blood vessels, distance between the top of lined capillary loops, and side and upper diameter of the capillary loop were manually measured using at least three loops. C: A capillaroscopic assessment method measuring the distance between each loop. The neighboring apex points were connected with a line for each distal loop. Loops with >90° angle were identified as top-lined capillaries and the distance (d1 and d2) was measured.

Since the fourth finger may get less damage by physical stress than the second and third fingers, the fourth finger of the left hand was selected for observation, and for any participant who had a problem with fourth finger used the fourth finger on their right hand. Nailfold capillaries were observed under a light microscope (Kekkan-Bijin, AT Co., Ltd., Osaka, Japan; region of observation 500 μm × 700 μm; magnification 320×, Fig 2A) after application of mineral oil to reduce light reflection. Capillary images were captured using Capillary Analysis System (AT Co., Ltd.).

### 2–4. Image analysis

The images of nailfold capillaries were numerically analyzed for four parameters: diameter, length, width, and distance. Major normal capillaroscopic pattern have been reported as a straight U-shaped and dense loops [28]. The twisted, bushy, non-dense, or small loops are commonly correlated with being unhealthy. One picture of nailfold capillaries was taken for each individual. In the pictures, side diameter of the capillary loop, length of the capillary loop, width of the parallel blood vessels, and distance between the top of lined capillary loops were manually measured using at least three loops (Fig 2B). Top-lined capillary loops were determined by previously described capillaroscopic assessment [29]. Briefly, in normal hairpin loop capillaries or complex capillaries, whose upper arches were bent or branching, apex points were identified at the midpoints of the upper arches or the top of the capillaries, respectively. The neighboring apex points were connected with a line for each distal loop. Loops with >90° angle were identified as top-lined capillaries and the distance was measured (Fig 2C). The mean value of these parameters for each individual was used for statistical analysis. Area and sums of capillary lengths were measured using the Capillary Analysis System.

### 2–5. Statistical analysis

Data were expressed as mean ± standard deviation. Two-tailed Student’s *t*-test or Welch’s *t*-test was used for single comparisons between sex or age. Correlation values (r values) were calculated for temperature of the fingertips, values of lifestyle habits, and parameters of nailfold capillaries; *t* values were then directly calculated using the r values and applied to *t* distribution to calculate *p* values. For the concern of multiple comparisons, the Bonferroni correction is performed under the condition that the correlations of each lifestyle habit were individually analyzed with the parameters of nailfold capillary (6 types). A statistically significant difference was defined as a *p* value of ≤0.05. Subsequently, principal component analysis (PCA) was performed using the r values for all data or data not including coldness, fingertip temperature, and parameters of area, and sums of capillary lengths [30].

### 2–6. Ethical consideration

This study was approved by the ethics committee of The University of Tokyo (approval number: 18–53 and 18–334). Written informed consent was obtained from each participant, and the participants can decline the test even after writing the informed consent. Upon collecting capillary pictures from participants, we anonymized by labeling the number.

## 3. Results

### 3–1. Relationship between the structures of capillaries and lifestyle habits

**Fig 3:**
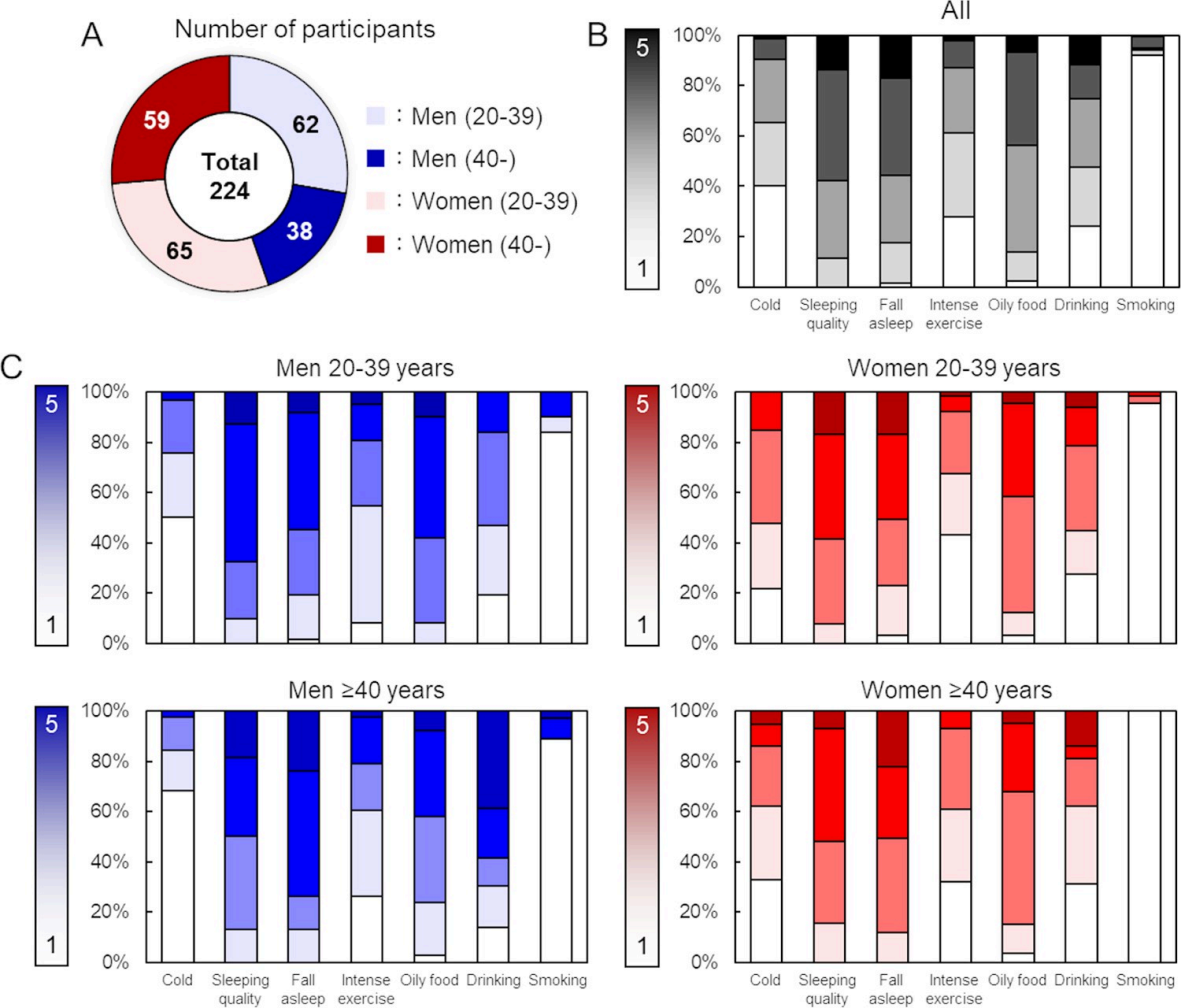
Characteristics of participants in the broad study. A: Distribution of sex and age in the broad study described in number of participants. B: Percentage of lifestyle habit scores in all participants. C: Percentage of lifestyle habit scores divided into men or women with aged 20–39 or ≥40 years. White indicated low scores, and black or dark color meant higher scores with gradient.

In the broad study, 224 individuals participated in nailfold capillary observations and completed the questionnaires; the size of the population was similar in men and women and participants aged ≥40 and <40 years (62 men aged 20–39 years, 38 men aged ≥40 years, 65 women aged 20–39 years, 59 women aged ≥40 years; Fig 3A). The scores of lifestyle habits are listed (Fig 3B and 3C; S2 Table in S1 File), and the distribution was wide for all parameters except for smoking, suggesting that the correlation between smoking and nailfold capillary structure is affected by bias.

**Fig 4:**
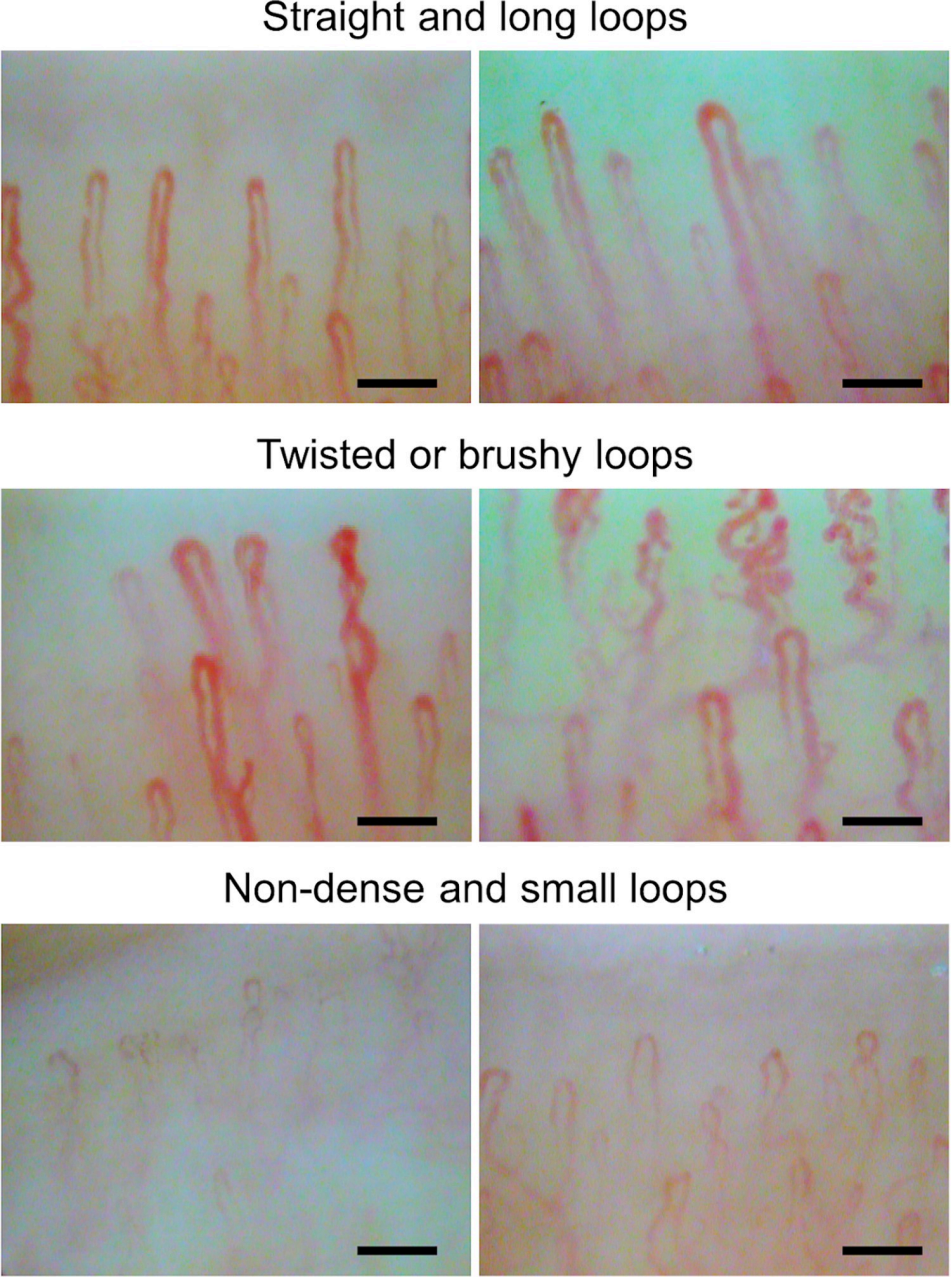
Representative pattern of nailfold capillaries. Representative images of nailfold capillaries showing straight and long loops, twisted or bushy loops, and non-dense and small loops. Scale bars: 100 μm.

In this study, various patterns were observed in the nailfold capillaroscopic images. For example, the straight and long loops were commonly correlated with being healthy (Fig 4A and 4B) and twisted or bushy loops (Fig 4C and 4D) and non-dense and small loops (Fig 4E and 4F) were commonly correlated with being unhealthy. However, any direct correlation of those abnormal loops with the lifestyle habit was not found.

**Fig 5:**
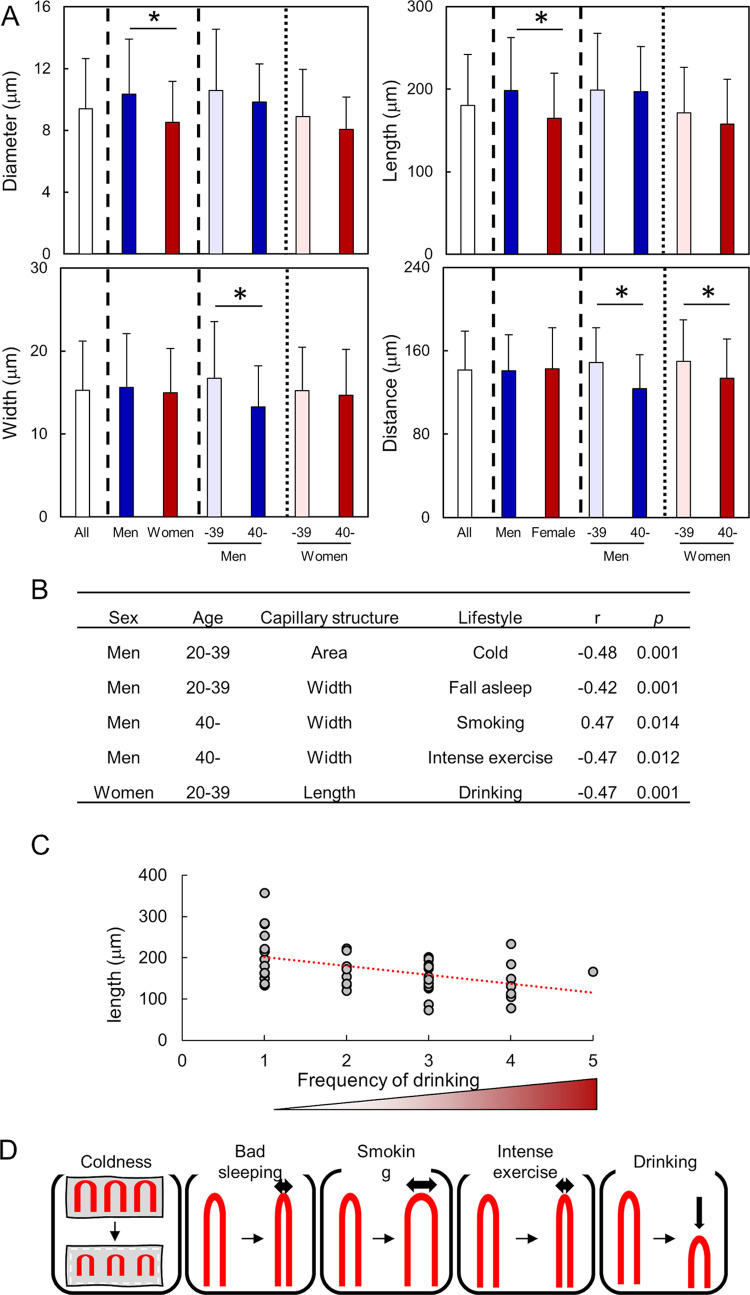
Structures of nailfold capillaries correlated with lifestyle habits. A: The mean values of parameters of nailfold capillaries in all participants, all men and women, and men and women aged 20–39 and ≥40 years. *: *p* ≤ 0.05. B: Statistically correlated parameters of nailfold capillary structure and lifestyle habits included the same group of principal component analysis. C: Representative dot plot showing length of the loop of nailfold capillaries negatively correlated with the frequency of drinking. Dashed line: least-squares line. D: Schematic representation of the correlation between the nailfold capillary structure and lifestyle habits.

To quantitively analyze various types of structures, the parameters of nailfold capillary were measured (Materials and Methods section and Fig 2). Side diameter of the capillary loop, length of the capillary loop, width of the parallel blood vessels, and distance between the top of lined capillary loops were 9.4 ± 3.3, 180 ± 62, 14.7 ± 5.9, and 142 ± 37 μm, respectively (Fig 5A). In women, the diameter and length were significantly lower than those in men. Furthermore, in men and women aged ≥40 years, the width and distance were significantly lower than that in those aged 20–39 years, except for the width in women. These data suggest that sex and age affect the pattern of nailfold capillaries and the effects mask the correlation of these factors with lifestyle habits. In fact, the r values were low when data from all individuals were analyzed (S3A Table in S1 File), and we could not find the same group of nailfold capillary structure and lifestyle habit with significant correlation by PCA (S3B Table in S1 File). Thus, these data were divided according to sexes and two age groups.

We evaluated the relationship between capillary structure and lifestyle habits using data in which the effects of sex and age were decreased. For men and women aged 20–39 and ≥40 years, the r values and results of PCA are listed in S4-S7 Tables of S1 File. PCA was performed in data including all or coldness- and temperature-removed data to detect a direct relationship between lifestyle habits and structures. Because parameters of total capillary length and area in the pictures strongly correlated with length, those were also removed from the analysis of coldness- and temperature-removed data for simplification. We focused on two factors for which lifestyle habit significantly correlated with nailfold capillary structure. The lifestyle habit and structure belonged to same group in each factor 1 or 2 by PCA to estimate a direct relationship (listed in Fig 5B). Capillary area negatively correlated with the coldness of fingertips in men aged 20–39 years. The width negatively correlated with falling asleep in men aged 20–39 years, and positively and negatively correlated with the frequency of smoking and intense exercise in men aged ≥40 years, respectively. In women, capillary length correlated with the frequency of drinking alcohol in those aged 20–39 year. For example, the dot plot indicated a tendency of small length of capillary loops in the high scoring group of drinking alcohol (Fig 5C). Fingertip temperatures did not significantly correlate with any parameters of capillary structure and lifestyle habits.

We found some relationships between the structures of capillaries and lifestyle habits from these results. The relationships indicated a possibility that poor sleeping habits, smoking, intense exercise, and drinking alcohol may cause changes in the pattern of nailfold capillaries (Fig 5D).

## 4. Discussion

In this study, we have evaluated the relationship between nailfold capillary pattern and lifestyle habits and coldness of the fingertips for routine health checkups with simple, inexpensive, and noninvasive methods. The frequencies of coldness of fingertips, falling asleep quickly, smoking, and intense exercise correlated with the nailfold capillary pattern depended on sex and age. Therefore, observation of the nailfold capillary pattern may give us suggestion for monitoring the lifestyle and routine health checkup.

In a previous review, the average diameter, length, width, and distance of the nailfold capillaries were reported to be 13, 190, 17, and 140 μm, respectively, in participants with non-Asian ethnicity [14]. The data is consistent with ours in this study except for the diameter; therefore, the structural parameters of nailfold capillaries are similar among different population groups. In the review, Korean ethnicity shows a lower diameter than that in European [14], supporting that Asian ethnicity has a lower diameter in this study. Regarding the structures of nailfold capillaries, individual differences and standard deviations were large both in this study and previous studies [14, 28]. Thus, a follow-up study may clarify the correlation between lifestyle habits and structures of the nailfold capillaries.

Studies have reported that plexus visibility scores in nailfold capillaries correlated with age and sex [31, 32]. Using a confocal laser-scanning microscope, capillary area and perimeter in the skin of the forearm was found to be constant within a narrow range, regardless of sex and age [33]. In this study, we found a strong correlation of sex and age with the structures of nailfold capillaries in a non-clinical population. The male participants and the participants aged 20–39 years had higher scores for all structural parameters compared with the female participants and the participants aged ≥40 years, respectively. At the cellular level, the density of total capillaries and functional capillaries covered by pericytes decrease with aging [34, 35]. In addition, aging directly affects signaling and the ability to form long capillaries in endothelial cells [36]. These reports support that aging may cause a decrease in capillary length and density in nailfold skin. For statistical analysis of the structures of nailfold capillaries, sex and age of the group should be divided to eliminate effects associated with sex and age.

The frequency of coldness of fingertips negatively correlated with the area of nailfold capillaries in young men. In this study, the reason or effect of coldness on capillary area is not distinguishable because coldness can directly cause damage to endothelial cells [37, 38] and blood flow transmits heat in the body. In women, sex hormones and menstrual cycle, including menopause, are related to body temperature [39]; therefore in men, the relationship between coldness and capillaries can be analyzed more simply than in women. Patients with Raynaud’s disease have a history of sensitivity to cold, episodes of symmetric pallor, and/or cyanosis of the fingertips after exposure to cold, and a close relationship with abnormal nailfold capillaries [12, 13]. Therefore, the coldness of fingertips was considered a pre-disease for Raynaud’s disease, and a small capillary area may be used to detect pre-disease for Raynaud’s phenomenon in young men. The frequencies of falling asleep quickly, smoking, and intense exercise correlated with the width of the nailfold capillary loops in men, and the frequency of drinking alcohol correlated with capillary length in women. Because the smoking population was small in women and long-term smoking may strongly affect circulation, the relationship between smoking and abnormal structures was detected only in older men. Psychological stress induced by poor sleep, smoking, and drinking alcohol and oxidative stress induced by intense exercise can change blood pressure, which are involved in cardiovascular phenotypes, such as hypertension and erectile dysfunction, especially in men [40, 41]. Furthermore, a change in pressure directly induces structural changes in nailfold capillaries, such as buckling, as described by the mathematical model [42], suggesting that these lifestyle factors affect physical parameters of blood flow and then change the width of nailfold capillary loops. Nicotine and ethanol disrupt the barrier function of endothelial cells, and nicotine inhibits the proliferation of endothelial cells [43–45]. Therefore, endothelial damage owing to cigarettes and alcohol can cause changes in the structures of nailfold capillaries.

We have evaluated whether observing their own capillaries promotes change in behavior, leading to structural changes in nailfold capillaries through improvement in lifestyle habits by the follow-up study (S1, S2 Figs in S1 File). After 1–2 weeks, the structural changes were detected in nailfold capillaroscopic images in some participants (S2 Fig in S1 File). The follow-up study may suggest that the observation of nailfold capillaries is one of the simple detection tools for health and may increase motivation for daily behavior changes in participants to improve their lifestyle habits. However, the sample size was not enough in the follow-up study and the significant correlations of lifestyle habits with the pattern of the nailfold capillaries were weak in broad studies. Therefore, the observation cannot be available to find the specific disease and lifestyle change yet.

In the limitation of this observation system, since the shapes of capillaries were detected by the red color derived from hemoglobin, the resolution of the nailfold capillaries have fluctuation among individuals with variations of hematocrit and hemoglobin [46] and even the same individual with a daily change [47]. Stable environmental parameters (e.g. schedule and temperature) are needed for the detection of differences in structures. The strength of this observation is the simple and easy method; however, some experiences for this observation are needed because of the variations in the depth and position of capillaries. Further, this observation system allows follow-up study by the detection of the same position of capillaries. To get an image of whole nailfold capillaries for detection of the same capillaries, the panorama observation system has been developed [13]. To detect the same position with no experience, we should develop a fully automated system searching a position, observing a panorama image, and analyzing the shapes of nailfold capillaries. Recently, the system converting 2D images to 3D objects without external supervision has been developed by machine learning [48]; therefore, 3D information for nailfold capillaries may be obtained by 2D images using a simple microscope system.

In conclusion, this study is the first report indicating a correlation between the structure of nailfold capillaries and lifestyle habits in a non-clinical population. The structure of nailfold capillaries correlated with coldness of fingertips, falling asleep, smoking, and intense exercise which were involved in cardiovascular phenotypes. The simple, inexpensive, and noninvasive method using nailfold microscopy can be employed for routine health checkups everywhere even at a bedside.

## Supporting information

S1 File (DOCX)
