# Supplementary material for: Nailfold capillary patterns correlate with age, gender, lifestyle habits, and fingertip temperature
Source: PLoS One. 2022 Jun 15;17(6):e0269661. doi: 10.1371/journal.pone.0269661 (PMC9200324; doi:10.1371/journal.pone.0269661)
Supplement: S1 File — (DOCX) [file pone.0269661.s001.docx]

**SUPPORTING INFORMATION**

**Nailfold capillary patterns correlate with lifestyle habits and fingertip temperature**

Authors: Tadaaki Nakajima^1¶*†^, Shizuka Nakano^1, 2¶^, Akihiko Kikuchi^2^, Yukiko T. Matsunaga^1*^.

1: Institute of Industrial Science, The University of Tokyo, 4-6-1 Komaba, Meguro-ku, Tokyo 153-8505, Japan

2: Department of Materials Science and Technology, Tokyo University of Science, 6-3-1 Niijuku, Katsushika-ku, Tokyo 125-8585, Japan

¶These authors have contributed equally to this work.

*Corresponding authors

†Current address: Department of Science, Yokohama City University, 22-2 Seto, Kanazawa-ku, Yokohama Kanagawa 236-0027, Japan. Tel.: +81-45-787–2271.

**SUPPORTING INFORMATION 1: SUPPORTING TABLES**

**Table S1** Lists of the questionnaires.

**Table S2** Detailed list of lifestyle habits in the broad study.

**Table S3** Correlation and PCA analysis in all participants of the broad study.

**Table S4** Correlation and PCA analysis in men aged 20-39 years in the broad study.

**Table S5** Correlation and PCA analysis in men aged ≥40 years in the broad study.

**Table S6** Correlation and PCA analysis in women aged 20-39 years in the broad study.

**Table S7** Correlation and PCA analysis in women aged ≥40 years in the broad study.

**SUPPORTING INFORMATION 2: FOLLOW-UP STUDY**

**1. Introduction**

**2. Materials and methods**

**2-1. Study design and participants**

**2-2. Questionnaires**

**2-3. Nailfold capillaroscopy, image analysis, and statistical analysis**

**2-4 Ethical consideration**

**3. Results**

**Figure S1** Frequency of full bathe correlated with the structure of nailfold capillary in the follow-up study.

**Figure S2** The improvement in lifestyle habits changed the structures of nailfold capillaries.

**SUPPORTING INFORMATION 1: SUPPORTING TABLES**

**Table S1** Lists of the questionnaires.

| Question | Type of answer |
| --- | --- |
| Gender | Men, Women, Unanswered |
| Age | Actual age |
| How often do you do feel coldness of fingertip? | Not at all, Rarely, Sometimes, Often, Always |
| How often do you experience good sleeping? | Little sleep, Sleep less, Sometimes sleep less, Sleep well, Sleep very well |
| How often do you fall asleep well? | Bad, Not so good, Quite a good, Good, Very good |
| How often do you do intense exercise? | Not at all, Once a month, Once a week, 2 or 3 times a week, Every day |
| How often do you consume oily food? | Not at all, Once a month, Once a week, 2 or 3 times a week, Every day |
| How often do you drink alcohol? | Not at all, Once a month, Once a week, 2 or 3 times a week, Every day |
| How often do you smoke? | Not at all, A little, Quite a lot, Less than one box every day, More than one box every day |
| How often do you take a full bath? (not just a shower) | Not at all, Sometimes, Every day |
| Do you want to improve lifestyle after observation of own capillary? | Yes or No |

**Table S2** Detailed list of lifestyle habits in the broad study.

| **Coldness of fingertip** | Degree | Men | | Women | |  |  |  | |  | |
| --- | --- | --- | --- | --- | --- | --- | --- | --- | --- | --- | --- |
|  |  | 20-39 | 40- | 20-39 | 40- |  |  |  |  |  |  |
| Not at all | 1 | 31 | 26 | 14 | 19 |  |  |  |  |  |  |
| Rarely | 2 | 16 | 6 | 17 | 17 |  |  |  |  |  |  |
| Sometimes | 3 | 13 | 5 | 24 | 14 |  |  |  |  |  |  |
| Often | 4 | 2 | 1 | 10 | 5 |  |  |  |  |  |  |
| Always | 5 | 0 | 0 | 0 | 3 |  |  |  |  |  |  |
|  |  | 62 | 38 | 65 | 58 |  |  |  |  |  |  |
| Total |  |  |  |  | 223 |  |  |  |  |  |  |
| **Sleep quality** | Degree | Men | | Women | | **Fall asleep** | Degree | Men | | Women | |
|  |  | 20-39 | 40- | 20-39 | 40- |  |  | 20-39 | 40- | 20-39 | 40- |
| Little sleep | 1 | 0 | 0 | 0 | 0 | Bad | 1 | 1 | 0 | 0 | 0 |
| Sleep less | 2 | 6 | 5 | 5 | 9 | Not so good | 2 | 11 | 5 | 5 | 9 |
| Sometimes sleep less | 3 | 14 | 14 | 22 | 19 | Quite a good | 3 | 16 | 5 | 22 | 19 |
| Sleep well | 4 | 34 | 12 | 27 | 26 | Good | 4 | 29 | 19 | 27 | 26 |
| Sleep very well | 5 | 8 | 7 | 11 | 4 | Very good | 5 | 5 | 9 | 11 | 4 |
|  |  | 62 | 38 | 65 | 58 |  |  | 62 | 38 | 65 | 58 |
| Total |  |  |  |  | 223 | Total |  |  |  |  | 223 |
| **Intense exercise** | Degree | Men | | Women | | **Oily food** | Degree | Men | | Women | |
|  |  | 20-39 | 40- | 20-39 | 40- |  |  | 20-39 | 40- | 20-39 | 40- |
| Not at all | 1 | 5 | 10 | 28 | 19 | Not at all | 1 | 0 | 1 | 2 | 2 |
| Once a month | 2 | 29 | 13 | 16 | 17 | Once a month | 2 | 5 | 8 | 6 | 7 |
| Once a week | 3 | 16 | 7 | 16 | 19 | Once a week | 3 | 21 | 13 | 30 | 31 |
| 2 or 3 times a week | 4 | 9 | 7 | 4 | 4 | 2 or 3 times a week | 4 | 30 | 13 | 24 | 16 |
| Every day | 5 | 3 | 1 | 1 | 0 | Every day | 5 | 6 | 3 | 3 | 3 |
|  |  | 62 | 38 | 65 | 59 |  |  | 62 | 38 | 65 | 59 |
| Total |  |  |  |  | 224 | Total |  |  |  |  | 224 |
| **Drinking alcohol** | Degree | Men | | Women | | **Smoking** | Degree | Men | | Women | |
|  |  | 20-39 | 40- | 20-39 | 40- |  |  | 20-39 | 40- | 20-39 | 40- |
| Not at all | 1 | 12 | 5 | 18 | 18 | Not at all | 1 | 52 | 32 | 61 | 58 |
| Once a month | 2 | 17 | 6 | 11 | 18 | A little | 2 | 4 | 0 | 0 | 0 |
| Once a week | 3 | 23 | 4 | 22 | 11 | Quite a lot | 3 | 0 | 0 | 2 | 0 |
| 2 or 3 times a week | 4 | 10 | 7 | 10 | 3 | Less than one box every day | 4 | 6 | 3 | 1 | 0 |
| Every day | 5 | 0 | 14 | 4 | 8 | More than one box every day | 5 | 0 | 1 | 0 | 0 |
|  |  | 62 | 36 | 65 | 58 |  |  | 62 | 36 | 64 | 58 |
| Total |  |  |  |  | 221 | Total |  |  |  |  | 220 |

**Table S3**  Correlation and PCA analysis in all participants of the broad study.

A: List of r values.

|  | Age | Cold | Sleeping | Fall asleep | Exercise | Oily food | Drinking | Smoking | Diameter | Length | Width | Distance | All length | Area |
| --- | --- | --- | --- | --- | --- | --- | --- | --- | --- | --- | --- | --- | --- | --- |
| Temp | 0.02 | -0.23 | 0.02 | -0.07 | -0.17 | -0.05 | 0.07 | -0.06 | -0.07 | -0.13 | -0.01 | 0.01 | -0.11 | 0.07 |
| Age |  | -0.07 | -0.13 | 0.12 | -0.04 | -0.15 | 0.18 | -0.06 | -0.14 | -0.10 | -0.13 | -0.27 | 0.16 | 0.19 |
| Cold |  |  | -0.09 | -0.01 | -0.10 | 0.00 | -0.10 | -0.09 | 0.07 | -0.03 | 0.06 | 0.05 | -0.07 | -0.13 |
| Sleeping |  |  |  | 0.45 | 0.12 | 0.09 | -0.04 | -0.11 | 0.06 | -0.04 | 0.09 | -0.02 | 0.04 | 0.03 |
| Fall asleep |  |  |  |  | 0.14 | 0.08 | 0.05 | -0.02 | 0.05 | -0.05 | 0.19 | -0.03 | 0.12 | 0.20 |
| Exercise |  |  |  |  |  | -0.05 | 0.07 | -0.01 | 0.00 | -0.04 | -0.08 | -0.06 | 0.10 | -0.06 |
| Oily food |  |  |  |  |  |  | -0.06 | 0.05 | 0.11 | 0.06 | 0.20 | -0.03 | -0.04 | -0.03 |
| Drinking |  |  |  |  |  |  |  | 0.18 | 0.01 | -0.02 | -0.02 | 0.05 | 0.07 | 0.00 |
| Smoking |  |  |  |  |  |  |  |  | 0.16 | 0.03 | 0.21 | -0.02 | -0.05 | 0.00 |
| Diameter |  |  |  |  |  |  |  |  |  | 0.23 | 0.08 | 0.19 | 0.06 | 0.10 |
| Length |  |  |  |  |  |  |  |  |  |  | -0.15 | 0.19 | 0.51 | 0.30 |
| Width |  |  |  |  |  |  |  |  |  |  |  | 0.10 | -0.13 | -0.03 |
| Distance |  |  |  |  |  |  |  |  |  |  |  |  | -0.22 | -0.20 |
| All length |  |  |  |  |  |  |  |  |  |  |  |  |  | 0.73 |
| Area |  |  |  |  |  |  |  |  |  |  |  |  |  |  |

Color-filled cells indicated a significant correlation (*p*≤0.05)

B: Results of PCA using all data.

|  | Factor1 | Factor2 | Factor3 | Factor4 | Factor5 | Factor6 |
| --- | --- | --- | --- | --- | --- | --- |
| All length | **0.89** | 0.04 | -0.23 | 0.05 | -0.28 | 0.17 |
| Area | **0.84** | 0.08 | -0.28 | -0.07 | -0.21 | 0.05 |
| Length | **0.77** | 0.02 | 0.00 | 0.33 | -0.07 | -0.01 |
| Distance | **0.51** | 0.11 | 0.34 | -0.33 | 0.31 | -0.15 |
| Temp | -0.07 | **-0.65** | -0.35 | 0.19 | 0.22 | -0.01 |
| Fall asleep | -0.23 | **0.64** | -0.57 | -0.06 | -0.09 | -0.17 |
| Cold | -0.13 | **-0.61** | -0.18 | -0.07 | -0.03 | -0.20 |
| Sleeping | -0.43 | **0.55** | -0.44 | -0.10 | -0.10 | -0.37 |
| Exercise | -0.21 | -0.18 | **-0.50** | -0.05 | 0.33 | 0.27 |
| Age | 0.16 | 0.30 | 0.37 | **-0.63** | 0.17 | 0.06 |
| Drinking | -0.28 | -0.28 | 0.25 | **-0.54** | -0.41 | 0.24 |
| Width | -0.17 | 0.41 | 0.23 | **0.46** | -0.15 | 0.37 |
| Smoking | -0.37 | -0.12 | 0.43 | 0.24 | **-0.60** | -0.15 |
| Diameter | 0.16 | 0.02 | 0.48 | 0.30 | 0.27 | **-0.63** |
| Oily food | -0.24 | 0.28 | 0.29 | 0.26 | 0.39 | **0.48** |

**Table S4**  Correlation and PCA analysis in men aged 20-39 years in the broad study.

A: List of r values.

|  | Cold | Sleeping | Fall asleep | Exercise | Oily food | Drinking | Smoking | Diameter | Length | Width | Distance | All length | Area |
| --- | --- | --- | --- | --- | --- | --- | --- | --- | --- | --- | --- | --- | --- |
| Temp | -0.07 | 0.22 | 0.10 | -0.13 | 0.09 | -0.15 | -0.12 | 0.02 | -0.12 | 0.05 | -0.05 | 0.02 | 0.14 |
| Cold |  | -0.07 | 0.10 | -0.14 | 0.22 | 0.07 | -0.02 | 0.21 | -0.11 | 0.21 | 0.13 | -0.43^*^ | -0.48^*^ |
| Sleeping |  |  | 0.21 | 0.18 | -0.01 | -0.26 | -0.21 | 0.19 | -0.20 | -0.02 | 0.03 | -0.21 | -0.05 |
| Fall asleep |  |  |  | 0.23 | -0.10 | 0.05 | -0.08 | 0.05 | -0.03 | 0.42^*^ | 0.08 | -0.10 | -0.03 |
| Exercise |  |  |  |  | -0.12 | -0.17 | -0.08 | -0.03 | -0.29 | -0.01 | -0.19 | 0.14 | 0.11 |
| Oily food |  |  |  |  |  | -0.22 | 0.07 | 0.06 | -0.08 | 0.23 | 0.06 | -0.06 | 0.08 |
| Drinking |  |  |  |  |  |  | 0.31 | 0.01 | 0.09 | -0.07 | 0.09 | -0.06 | -0.22 |
| Smoking |  |  |  |  |  |  |  | 0.17 | -0.10 | 0.22 | -0.04 | -0.19 | -0.12 |
| Diameter |  |  |  |  |  |  |  |  | 0.04 | 0.13 | 0.23 | -0.23 | -0.21 |
| Length |  |  |  |  |  |  |  |  |  | -0.25 | 0.19 | 0.47 | 0.25 |
| Width |  |  |  |  |  |  |  |  |  |  | 0.08 | -0.26 | -0.04 |
| Distance |  |  |  |  |  |  |  |  |  |  |  | -0.17 | -0.21 |
| All length |  |  |  |  |  |  |  |  |  |  |  |  | 0.79 |
| Area |  |  |  |  |  |  |  |  |  |  |  |  |  |

Color-filled cells indicated a significant correlation (*p*≤0.05)

*: *p*≤0.05 with Bonferroni correction

B: Results of PCA using all data.

|  | Factor1 | Factor2 | Factor3 | Factor4 | Factor5 | Factor6 |
| --- | --- | --- | --- | --- | --- | --- |
| All length | **0.88** | -0.14 | -0.01 | -0.16 | -0.37 | 0.00 |
| Area | **0.86** | -0.26 | -0.13 | 0.13 | -0.20 | -0.11 |
| Cold | **0.72** | 0.19 | -0.14 | 0.27 | -0.06 | -0.33 |
| Width | -0.02 | **0.80** | -0.03 | -0.26 | -0.37 | 0.15 |
| Fall asleep | -0.36 | **0.60** | 0.31 | -0.34 | -0.13 | -0.20 |
| Sleeping | -0.37 | **-0.52** | 0.00 | -0.43 | -0.12 | -0.26 |
| Drinking | -0.41 | **-0.42** | -0.34 | 0.39 | -0.23 | 0.29 |
| Exercise | -0.15 | -0.24 | **0.74** | 0.15 | 0.08 | 0.14 |
| Smoking | -0.32 | -0.21 | **-0.56** | -0.29 | -0.28 | 0.40 |
| Length | 0.38 | -0.24 | **0.53** | -0.15 | -0.41 | 0.31 |
| Oily food | -0.16 | 0.45 | -0.15 | **0.72** | -0.04 | 0.08 |
| Distance | 0.32 | -0.04 | 0.24 | 0.00 | **0.69** | 0.38 |
| Diameter | 0.33 | 0.04 | -0.45 | -0.42 | **0.57** | -0.07 |
| Temp | -0.38 | -0.34 | 0.08 | 0.16 | -0.05 | **-0.61** |

C: Results of PCA using all data not including coldness, the temperature of fingertips, and parameters of the area and all summed length values of the capillaries.

|  | Factor1 | Factor2 | Factor3 |
| --- | --- | --- | --- |
| Width | **0.81** | -0.32 | -0.05 |
| Sleeping | **0.60** | -0.15 | -0.29 |
| Fall asleep | **-0.60** | -0.13 | -0.09 |
| Drinking | **-0.58** | -0.48 | -0.28 |
| Oily food | **0.33** | -0.27 | -0.21 |
| Distance | 0.02 | **0.67** | 0.40 |
| Smoking | -0.35 | **-0.63** | 0.17 |
| Length | -0.05 | **0.52** | -0.36 |
| Diameter | -0.03 | 0.08 | **0.89** |
| Exercise | -0.22 | 0.55 | **-0.57** |

**Table S5**  Correlation and PCA analysis in men aged ≥40 years in the broad study.

A: List of r values.

|  | Cold | Sleeping | Fall asleep | Exercise | Oily food | Drinking | Smoking | Diameter | Length | Width | Distance | All length | Area |
| --- | --- | --- | --- | --- | --- | --- | --- | --- | --- | --- | --- | --- | --- |
| Temp | -0.05 | 0.19 | 0.02 | -0.23 | 0.05 | -0.10 | -0.15 | 0.12 | -0.13 | -0.04 | -0.21 | -0.01 | 0.27 |
| Cold |  | -0.33 | -0.24 | 0.00 | 0.05 | -0.14 | 0.07 | 0.12 | 0.09 | -0.09 | 0.10 | 0.10 | 0.37 |
| Sleeping |  |  | 0.49 | 0.08 | 0.03 | 0.24 | -0.29 | 0.02 | -0.06 | 0.08 | -0.08 | 0.08 | -0.06 |
| Fall asleep |  |  |  | 0.13 | 0.13 | 0.08 | -0.02 | 0.29 | -0.01 | 0.22 | 0.30 | 0.26 | 0.24 |
| Exercise |  |  |  |  | -0.25 | -0.10 | -0.22 | -0.18 | -0.12 | -0.47 | -0.01 | 0.01 | -0.28 |
| Oily food |  |  |  |  |  | 0.02 | -0.09 | 0.37 | 0.13 | -0.15 | 0.09 | -0.14 | -0.06 |
| Drinking |  |  |  |  |  |  | 0.13 | -0.07 | -0.05 | 0.12 | 0.11 | 0.14 | -0.16 |
| Smoking |  |  |  |  |  |  |  | 0.24 | -0.01 | 0.47 | 0.04 | -0.05 | -0.01 |
| Diameter |  |  |  |  |  |  |  |  | 0.47 | 0.34 | 0.29 | 0.26 | 0.54 |
| Length |  |  |  |  |  |  |  |  |  | -0.08 | 0.04 | 0.78 | 0.70 |
| Width |  |  |  |  |  |  |  |  |  |  | 0.26 | -0.04 | 0.19 |
| Distance |  |  |  |  |  |  |  |  |  |  |  | -0.09 | 0.01 |
| All length |  |  |  |  |  |  |  |  |  |  |  |  | 0.69 |
| Area |  |  |  |  |  |  |  |  |  |  |  |  |  |

Color-filled cells indicated a significant correlation (*p*≤0.05)

B: Results of PCA using all data.

|  | Factor1 | Factor2 | Factor3 | Factor4 | Factor5 | Factor6 |
| --- | --- | --- | --- | --- | --- | --- |
| Length | 0.95 | 0.03 | 0.14 | -0.07 | -0.08 | 0.05 |
| Area | 0.91 | -0.06 | 0.16 | -0.16 | -0.22 | -0.10 |
| All length | 0.90 | -0.17 | 0.03 | -0.13 | -0.04 | 0.14 |
| Diameter | 0.58 | 0.45 | -0.42 | -0.03 | -0.13 | 0.08 |
| Smoking | -0.52 | 0.41 | 0.21 | -0.40 | -0.22 | 0.15 |
| Exercise | -0.19 | 0.68 | 0.24 | -0.20 | -0.02 | -0.34 |
| Width | -0.36 | 0.68 | -0.17 | -0.38 | -0.35 | 0.16 |
| Sleeping | -0.50 | -0.66 | 0.00 | -0.28 | -0.03 | -0.23 |
| Cold | 0.02 | -0.63 | -0.07 | -0.30 | -0.05 | -0.24 |
| Fall asleep | -0.13 | -0.45 | -0.67 | -0.22 | -0.14 | -0.20 |
| Temp | -0.12 | 0.04 | 0.49 | 0.66 | -0.31 | -0.39 |
| Distance | -0.22 | -0.03 | -0.59 | 0.60 | -0.33 | 0.31 |
| Oily food | 0.10 | 0.49 | -0.36 | 0.10 | 0.73 | -0.21 |
| Drinking | -0.23 | -0.34 | 0.42 | -0.03 | 0.26 | 0.64 |

C: Results of PCA using all data not including coldness, the temperature of fingertips, and parameters of the area and all summed length values of the capillaries.

|  | Factor1 | Factor2 | Factor3 |
| --- | --- | --- | --- |
| Fall asleep | **-0.84** | 0.02 | 0.00 |
| Diameter | **0.72** | -0.38 | -0.21 |
| Oily food | **0.57** | -0.19 | 0.01 |
| Length | **0.54** | -0.47 | 0.43 |
| Smoking | 0.01 | **0.77** | -0.05 |
| Width | 0.34 | **0.71** | -0.47 |
| Exercise | 0.39 | **0.64** | -0.01 |
| Distance | -0.09 | -0.39 | **-0.66** |
| Sleeping | -0.51 | -0.33 | **-0.59** |
| Drinking | -0.52 | 0.09 | **0.54** |

**Table S6**  Correlation and PCA analysis in women aged 20-39 years in the broad study.

A: List of r values.

|  | Cold | Sleeping | Fall asleep | Exercise | Oily food | Drinking | Smoking | Diameter | Length | Width | Distance | All length | Area |
| --- | --- | --- | --- | --- | --- | --- | --- | --- | --- | --- | --- | --- | --- |
| Temp | -0.21 | 0.03 | -0.07 | -0.26 | -0.05 | 0.19 | 0.06 | -0.15 | -0.07 | -0.01 | 0.20 | -0.01 | 0.20 |
| Cold |  | 0.14 | 0.09 | -0.04 | -0.25 | -0.01 | -0.19 | 0.14 | 0.16 | -0.04 | -0.16 | 0.29 | 0.01 |
| Sleeping |  |  | 0.57 | 0.08 | 0.01 | -0.04 | 0.09 | -0.11 | 0.05 | 0.19 | 0.05 | 0.24 | 0.11 |
| Fall asleep |  |  |  | 0.07 | 0.03 | -0.05 | 0.06 | 0.19 | -0.02 | 0.21 | -0.01 | 0.21 | 0.32 |
| Exercise |  |  |  |  | 0.11 | 0.15 | 0.15 | -0.15 | -0.07 | -0.09 | -0.02 | -0.10 | -0.28 |
| Oily food |  |  |  |  |  | 0.15 | 0.11 | -0.11 | 0.04 | 0.22 | -0.03 | -0.10 | -0.07 |
| Drinking |  |  |  |  |  |  | 0.13 | -0.06 | -0.47^*^ | 0.18 | 0.12 | -0.37 | -0.26 |
| Smoking |  |  |  |  |  |  |  | -0.08 | 0.07 | 0.11 | -0.05 | -0.03 | 0.05 |
| Diameter |  |  |  |  |  |  |  |  | 0.15 | -0.19 | 0.20 | 0.27 | 0.27 |
| Length |  |  |  |  |  |  |  |  |  | -0.17 | 0.28 | 0.38 | 0.05 |
| Width |  |  |  |  |  |  |  |  |  |  | 0.10 | -0.23 | -0.01 |
| Distance |  |  |  |  |  |  |  |  |  |  |  | -0.04 | -0.06 |
| All length |  |  |  |  |  |  |  |  |  |  |  |  | 0.58 |
| Area |  |  |  |  |  |  |  |  |  |  |  |  |  |

Color-filled cells indicated a significant correlation (*p*≤0.05)

*: *p*≤0.05 with Bonferroni correction

B: Results of PCA using all data.

|  | Factor1 | Factor2 | Factor3 | Factor4 | Factor5 | Factor6 |
| --- | --- | --- | --- | --- | --- | --- |
| Fall asleep | **0.89** | -0.18 | 0.04 | 0.13 | -0.15 | -0.11 |
| Sleeping | **0.78** | -0.26 | 0.22 | 0.16 | -0.23 | -0.26 |
| Drinking | -0.27 | **0.77** | 0.03 | -0.30 | -0.24 | -0.17 |
| All length | 0.39 | **0.75** | 0.12 | -0.03 | 0.42 | -0.15 |
| Length | -0.36 | **0.70** | 0.38 | 0.14 | -0.18 | -0.16 |
| Smoking | -0.24 | **-0.40** | 0.06 | -0.35 | -0.12 | -0.37 |
| Exercise | -0.05 | -0.06 | **-0.73** | -0.38 | -0.15 | 0.12 |
| Temp | -0.36 | -0.13 | **-0.66** | 0.27 | -0.07 | -0.05 |
| Width | 0.18 | 0.01 | **0.57** | -0.31 | -0.25 | 0.51 |
| Area | 0.48 | 0.51 | **-0.55** | -0.06 | 0.27 | -0.01 |
| Distance | -0.48 | 0.04 | 0.13 | **0.70** | -0.22 | 0.17 |
| Oily food | -0.27 | -0.25 | 0.25 | **-0.51** | 0.48 | 0.28 |
| Cold | -0.24 | -0.30 | 0.25 | 0.25 | **0.67** | -0.39 |
| Diameter | 0.20 | 0.11 | -0.11 | 0.38 | 0.30 | **0.63** |

C: Results of PCA using all data not including coldness, the temperature of fingertips, and parameters of the area and all summed length values of the capillaries.

|  | Factor1 | Factor2 | Factor3 | Factor4 |
| --- | --- | --- | --- | --- |
| Sleeping | **0.86** | -0.29 | -0.23 | -0.03 |
| Fall asleep | **0.83** | -0.31 | -0.29 | -0.12 |
| Length | **-0.70** | -0.50 | -0.29 | -0.05 |
| Drinking | **-0.67** | -0.12 | -0.57 | 0.01 |
| Exercise | -0.02 | **0.56** | 0.08 | -0.39 |
| Distance | -0.41 | **-0.55** | 0.47 | -0.12 |
| Smoking | -0.05 | **0.53** | -0.07 | -0.42 |
| Diameter | 0.11 | -0.22 | **0.75** | 0.10 |
| Width | 0.14 | -0.04 | -0.15 | **0.75** |
| Oily food | -0.15 | 0.57 | 0.07 | **0.63** |

**Table S7**  Correlation and PCA analysis in women aged ≥40 years in the broad study.

A: List of r values.

|  | Cold | Sleeping | Fall asleep | Exercise | Oily food | Drinking | Diameter | Length | Width | Distance | All length | Area |
| --- | --- | --- | --- | --- | --- | --- | --- | --- | --- | --- | --- | --- |
| Temp | -0.47 | -0.21 | -0.26 | -0.06 | -0.19 | 0.17 | -0.24 | -0.30 | -0.06 | -0.08 | -0.33 | -0.18 |
| Cold |  | -0.22 | 0.01 | 0.05 | 0.11 | -0.07 | 0.06 | 0.09 | 0.05 | 0.02 | 0.10 | 0.05 |
| Sleeping |  |  | 0.62 | 0.10 | 0.25 | -0.08 | 0.05 | 0.06 | 0.13 | -0.21 | 0.18 | 0.21 |
| Fall asleep |  |  |  | 0.17 | 0.35 | 0.00 | -0.11 | -0.03 | -0.02 | -0.19 | 0.19 | 0.27 |
| Exercise |  |  |  |  | -0.14 | 0.24 | 0.06 | 0.15 | -0.10 | -0.10 | 0.26 | 0.18 |
| Oily food |  |  |  |  |  | -0.20 | 0.13 | 0.08 | 0.22 | -0.40 | 0.05 | -0.09 |
| Drinking |  |  |  |  |  |  | 0.02 | 0.11 | -0.08 | 0.16 | 0.22 | 0.15 |
| Diameter |  |  |  |  |  |  |  | 0.35 | -0.01 | 0.00 | 0.11 | -0.04 |
| Length |  |  |  |  |  |  |  |  | -0.15 | 0.22 | 0.51 | 0.24 |
| Width |  |  |  |  |  |  |  |  |  | -0.15 | 0.10 | -0.11 |
| Distance |  |  |  |  |  |  |  |  |  |  | -0.44 | -0.31 |
| All length |  |  |  |  |  |  |  |  |  |  |  | 0.77 |
| Area |  |  |  |  |  |  |  |  |  |  |  |  |

B: Results of PCA using all data.

|  | Factor1 | Factor2 | Factor3 | Factor4 | Factor5 | Factor6 |
| --- | --- | --- | --- | --- | --- | --- |
| All length | **-0.85** | 0.28 | 0.40 | 0.08 | 0.10 | -0.01 |
| Length | **-0.71** | -0.32 | 0.12 | -0.39 | -0.02 | 0.05 |
| Area | **-0.66** | 0.46 | 0.44 | 0.12 | 0.07 | -0.12 |
| Sleeping | **0.58** | 0.38 | 0.57 | 0.06 | -0.09 | -0.18 |
| Oily food | **0.56** | 0.38 | -0.20 | -0.26 | 0.19 | 0.47 |
| Distance | -0.24 | **0.67** | 0.03 | -0.23 | 0.39 | 0.36 |
| Temp | 0.05 | **-0.62** | 0.53 | 0.20 | 0.24 | 0.17 |
| Cold | 0.27 | **-0.62** | 0.26 | 0.41 | 0.41 | -0.08 |
| Diameter | -0.08 | **-0.59** | 0.06 | -0.57 | -0.44 | 0.21 |
| Fall asleep | 0.50 | 0.47 | **0.59** | -0.05 | -0.32 | -0.01 |
| Drinking | -0.19 | 0.08 | -0.47 | **0.57** | 0.01 | 0.44 |
| Exercise | -0.20 | 0.22 | -0.36 | 0.42 | **-0.60** | -0.15 |
| Width | 0.09 | 0.15 | -0.52 | -0.30 | 0.41 | **-0.60** |

C: Results of PCA using all data not including coldness, the temperature of fingertips, and parameters of the area and all summed length values of the capillaries.

|  | Factor1 | Factor2 | Factor3 | Factor4 |
| --- | --- | --- | --- | --- |
| Sleeping | **0.84** | -0.41 | -0.23 | -0.01 |
| Fall asleep | **0.82** | -0.34 | -0.17 | 0.15 |
| Length | **-0.68** | -0.35 | 0.29 | -0.15 |
| Oily food | **0.56** | 0.28 | 0.39 | -0.30 |
| Diameter | -0.50 | **-0.71** | 0.10 | -0.20 |
| Drinking | -0.23 | **0.65** | -0.36 | -0.33 |
| Exercise | -0.19 | 0.31 | **-0.72** | 0.05 |
| Distance | 0.33 | 0.37 | **0.56** | -0.44 |
| Width | -0.09 | 0.37 | 0.45 | **0.81** |

**SUPPORTING INFORMATION 2: FOR FOLLOW-UP STUDY**

**1. Introduction**

We have evaluated whether observing their own capillaries promotes change in behavior, leading to structural changes in nailfold capillaries through improvement in lifestyle habits by the follow-up study.

**2. Materials and methods**

**2-1. Study design and participants**

***Follow-up study:*** We recruited 48 participants (Japanese women aged 21-66 years) by snowball sampling in 2019 February. Because we planned to evaluate whether observing their own capillaries promotes change in participant behavior, we recruited them by voluntary response. The room temperature was set at 25℃. Questionnaires, finger temperatures, and images of nailfold capillaries were evaluated by the methods described for the first test. During the first test, we explained participants following examples of good lifestyle habits to increase systemic circulation; routine dieting, moderate exercising, taking full baths, decreasing psychological stress by taking good sleep, decreasing alcohol intake, and increasing intake of foods to protect and maintain the function of endothelial cells. After observing their capillaries microscopically, the participants spontaneously selected which they improved their lifestyle habits or not and decided the methods. After 1–2 weeks, 11 participants opted to observe their nailfold capillaries and answered the questionnaires of the second test.

**2-2. Questionnaires**

In addition to the questionnaire of the broad study, two questions, including (i) frequency of full bath (1–3) and (ii) “Do you want to improve lifestyle after observation of own capillaries” (yes or no), were added to the first test. In the second test, we added details on improvement in lifestyle habits to the questionnaires. A list of the questions from both studies is presented in Table S1.

**2-3. Nailfold capillaroscopy, image analysis, and statistical analysis**

In the follow-up study, the participants were exposed to a comfortable temperature (25°C) at least 10 min before they observed their nailfold capillaries. Three images were taken and at least nine capillaries were evaluated for each individual, and diameters were measured as top, side, small, and large (Fig. 2B). The detailed methods for nailfold capillaroscopy, image analysis, and statistical analysis is described in the main manuscripts.

**2-4 Ethical consideration**

This study was approved by the ethics committee of The University of Tokyo (approval number: 18-53 and 18-334). Written informed consent was obtained from each participant, and the participants can decline the test even after writing the informed consent. Upon collecting capillary pictures from participants, we anonymized by labeling the number.

**3. Results**

**Time-dependent structural changes in nailfold capillaries**

We preliminarily investigated the extent to which structures of nailfold capillaries change after a conscious improvement in lifestyle habits. In the follow-up study at first observation, we introduced good lifestyle habits for improving circulation and blood vessel health (intake of healthy diet, decrease the frequency of drinking alcohol, application of moderate exercise, take full baths (Fig. S1), and good sleep) to the participants. For 1–2 weeks after first test, some participants improved their lifestyle habits voluntarily and opted to observe their nailfold capillaries in the second test. In the second test, all participants had improved their lifestyle habits.

After 1–2 weeks, the structural changes were detected in nailfold capillaroscopic images. In two examples from the follow-up study, meandering nailfold capillaries had straightened hairpin loops with the improvement in lifestyle (Fig. S2). Only 11 participants were detected in the second test (statistical power=0.43); therefore, we could not statistically analyze the data.

**Supporting Figures**


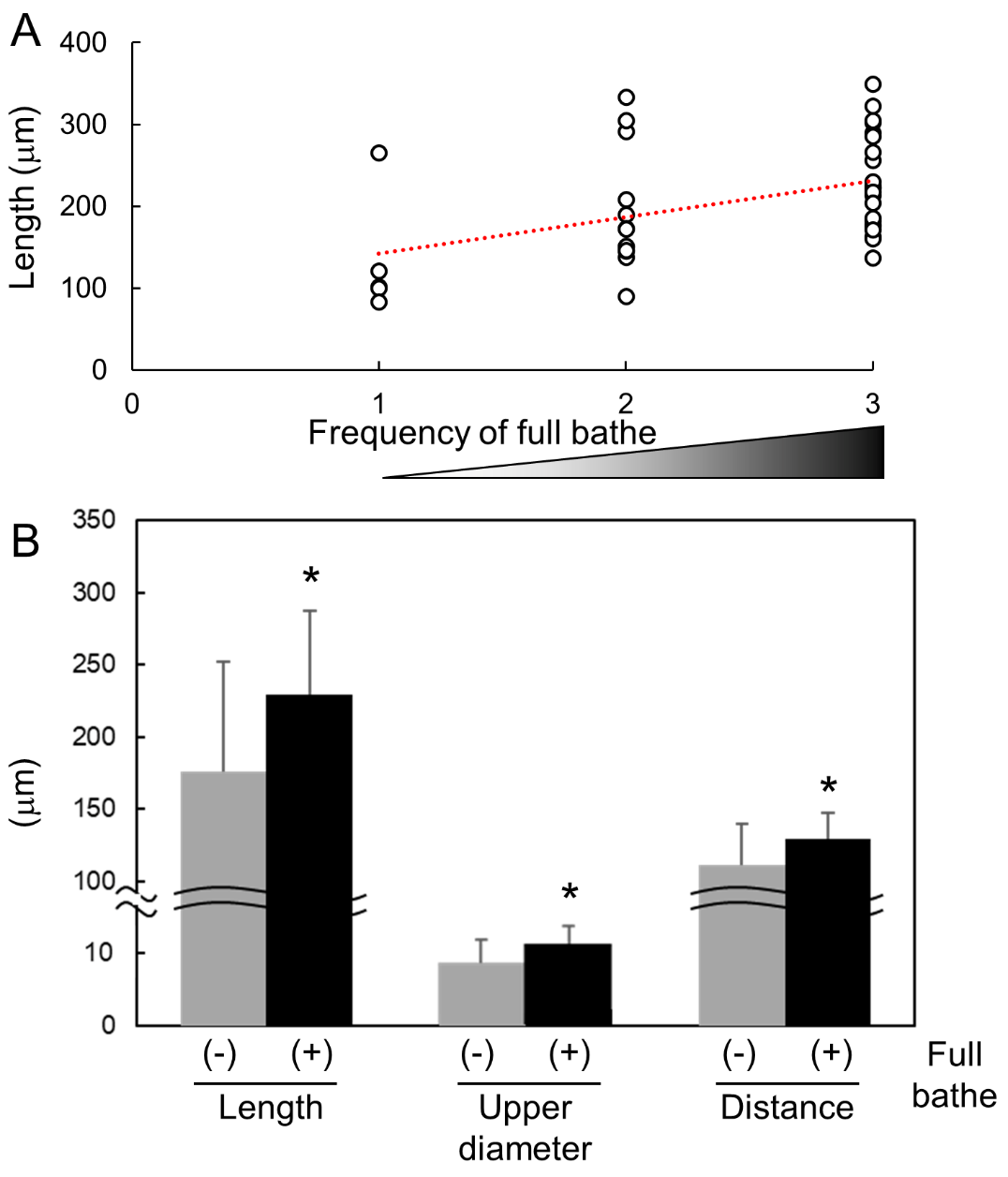


**Figure S1 Frequency of full bathe correlated with the structure of nailfold capillary in the follow-up study.**

A: Example of dot plots in which length of the loop of nailfold capillaries positively correlated with the score of the frequency of full bathe. Dashed line: least-squares line. B: parameters of nailfold capillaries expressing a significant difference between groups divided to individuals having no full bathe and sometimes or always having a full bathe. *: *p*≤0.05.


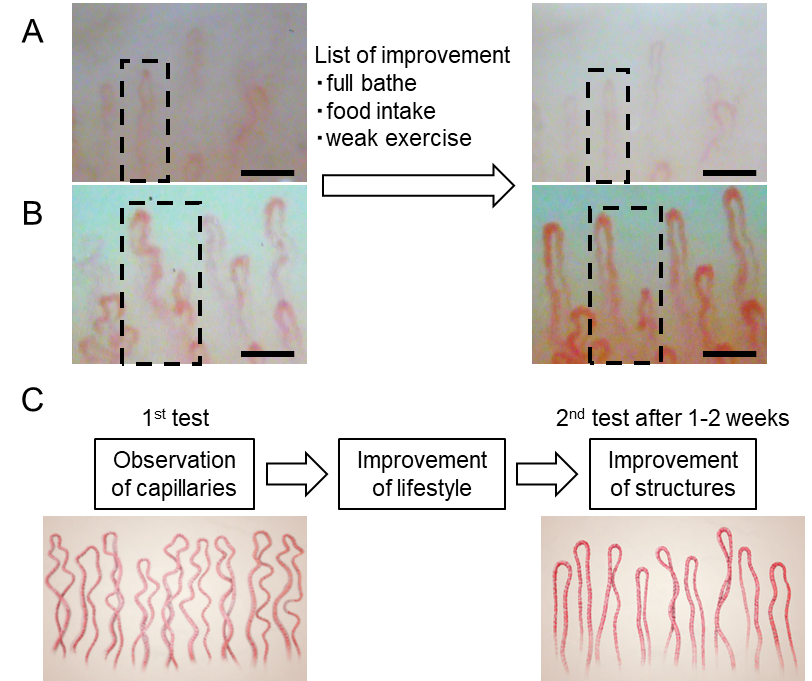


**Figure S2 The improvement in lifestyle habits changed the structures of nailfold capillaries.**

A, B: Two examples of nailfold capillary images at the first and second test. The improved lifestyle habits included increase in the frequency of full baths, less strenuous exercise, and consuming red wine or olive oil. Dashed boxes indicate the same loop. Scale bars: 100 μm. C: According to our hypothesis, observation of nailfold capillaries causes an improvement in lifestyle habits, and thus improves the structure of the nailfold capillaries.
